# Typhlitis, Perityphlitis, and Appendicitis

**Published:** 1891-03-14

**Authors:** 


### SURGERY.

### TYPHLITIS, PERITYPHLITIS, AND APPENDICITIS.

The pathology and diagnosis of these affections, although by no means definitely settled, have been made much clearer by recent research, and it is to be hoped that the rules of treatment will soon be placed on a more satisfactory basis. At present, however, the question of operative interference, its advisability, its method, and its results is unsettled. As the vermiform appendix is the cause of much trouble and danger, it is well that its normal relations and position should be understood. Dr. Fergusson gives the results of the examination of two hundred bodies, and some important points regarding this rudimentary organ are indicated. Its average length was found to be four and a-half inches, and its diameter that of a No. 9 English catheter. In the majority of cases in was situated behind the cæcum; in 123 specimens there was a distinct mesentery, and in these it would open (in case of perforation) into the peritoneal cavity. But in 77 subjects it was extra peritoneal, and this fact would alter the symptoms and prognosis in cases of perforation, inasmuch as the fæcal matter would escape into a less fatal tissue. The pus formed in such cases would follow the course of a psoas abcess and point in the groin. In seven specimens there was evidence of old lesions, and in three there were actual proofs of former perforation. In fifteen appendices foreign bodies were found, chiefly pieces of bone, orange pips, cherry stones, and in one case a screw.

Dr. Talamore recognises four varieties of inflammation of the appendix, viz: (1) acute perforating appendicitis; (2) subacute appendicitis with slowly occurring perforation; (3) simple appendicitis with colic; (4) relapsing appendicitis. He states (and it is obvious) that it is impossible to predict in any case whether perforation is going to occur; when it has occurred it can only be definitely diagnosed in the acute forms. In recurring cases the surroundings are so altered that the event produces modified symptoms, and in those cases mentioned above in which the appendix was found by Dr. Fergusson to be extra peritoneal the signs would be much less severe.

Haussman, criticising a work by Dennis, lays stress on the broad distinction, from a pathological view, between appendicitis and periappendicitis on the one hand, and typhlitis and perityphlitis on the other. Typhlitis is, he says. catarrhal or ulcerative inflammation of one or all the coats of the cæcum; it follows fæcal engorgement, septic diarrhœa, the action of corrosives, or some ulcerative process. Perityphlitis may follow this or may be primary, as in a case recorded of injury to the ilio-psoas from violent gymnastic exercise. He points out that the diagnosis of appendicitis may be confounded with renal colic, ovarian disease, intestinal obstruction, pyelitis, caries of the ilium, abscess of the liver, &c., &c. Although the difficulty of diagnosis is somewhat exaggerated by him, yet this list clearly shows that there is much obscurity still attending these affections, and no doubt mistakes are sometimes unavoidable.

Dr. Dodge, of Michigan, lays down the rule that when the symptoms are acute and violent abdominal section should be performed. In this most surgeons will agree. When he goes on to state, however, that these cases should first be treated with frequent doses of sulphate of magnesia, and large warm water enemata, there is no doubt that many will strongly disagree with him. Yet his cases seem to bear out his views. It is doubtful whether any real h rm can be done by mild purgatives beyond loss of time, and it is only in cases where the symptoms are not very pronounced that he seems to inculcate this line of treatment. He distinctly urges lateral abdominal section if there is no speedy relief. If an abscess is formed the contents should (he thinks) be evacuated and the cavity washed out with sterilised (boiled) water; the abscess walls should be stitched to the parietal peritoneum, and a drainage tube inserted. The after treatment should consist of daily irrigation of the cavity.

Considering the acknowledged obscurity of an early diagnosis it certainly appears somewhat rash to operate before trying milder measures, but unfortunately a few hours may decide the fate of the patient, and there is much to be said in favour of the conclusions arrived at by Mr. Weir, who in a paper read before the Medical Society of New York says, "all use of purgatives or enemata *in the beginning* should be avoided." He urges immediate operation as soon as there are signs of pus, or if symptoms of peritonitis increase. He considers the risk of the disease greater than the risk of a laparotomy.

On the whole we may state that the result of post-mortem examinations and the statistics of operations indicate that each case must be treated, not merely on the diagnosis, but on the condition of the patient, and on the signs of pus formation and peritonitis. It would be rash to operate solely because there is an appendicitis or a perityphlitis; and it would be dangerous to delay if there are distinct evidences of peritonitis or the formation of an abscess. The number of carefully recorded abdominal sections in these cases is too scanty to form any dogmatic rules, and when recovery has followed palliative treatment it is not always easy to believe that the symptoms have been indicative of more than localised and temporary inflammation of the walls of the intestine.

